# Comprehensive analysis of nicotinamide metabolism-related signature for predicting prognosis and immunotherapy response in breast cancer

**DOI:** 10.3389/fimmu.2023.1145552

**Published:** 2023-03-08

**Authors:** Hanxiao Cui, Xueting Ren, Luyao Dai, Lidan Chang, Dandan Liu, Zhen Zhai, Huafeng Kang, Xiaobin Ma

**Affiliations:** Department of Oncology, The Second Affiliated Hospital of Xi’an Jiaotong University, Xi’an, Shaanxi, China

**Keywords:** breast cancer, nicotinamide metabolism, prognosis, tumor microenvironment, immunotherapy

## Abstract

**Background:**

Breast cancer (BC) is the most common malignancy among women. Nicotinamide (NAM) metabolism regulates the development of multiple tumors. Herein, we sought to develop a NAM metabolism-related signature (NMRS) to make predictions of survival, tumor microenvironment (TME) and treatment efficacy in BC patients.

**Methods:**

Transcriptional profiles and clinical data from The Cancer Genome Atlas (TCGA) were analyzed. NAM metabolism-related genes (NMRGs) were retrieved from the Molecular Signatures Database. Consensus clustering was performed on the NMRGs and the differentially expressed genes between different clusters were identified. Univariate Cox, Lasso, and multivariate Cox regression analyses were sequentially conducted to develop the NAM metabolism-related signature (NMRS), which was then validated in the International Cancer Genome Consortium (ICGC) database and Gene Expression Omnibus (GEO) single-cell RNA-seq data. Further studies, such as gene set enrichment analysis (GSEA), ESTIMATE, CIBERSORT, SubMap, and Immunophenoscore (IPS) algorithm, cancer-immunity cycle (CIC), tumor mutation burden (TMB), and drug sensitivity were performed to assess the TME and treatment response.

**Results:**

We identified a 6-gene NMRS that was significantly associated with BC prognosis as an independent indicator. We performed risk stratification according to the NMRS and the low-risk group showed preferable clinical outcomes (*P* < 0.001). A comprehensive nomogram was developed and showed excellent predictive value for prognosis. GSEA demonstrated that the low-risk group was predominantly enriched in immune-associated pathways, whereas the high-risk group was enriched in cancer-related pathways. The ESTIMATE and CIBERSORT algorithms revealed that the low-risk group had a higher abundance of anti-tumor immunocyte infiltration (*P* < 0.05). Results of Submap, IPS, CIC, TMB, and external immunotherapy cohort (iMvigor210) analyses showed that the low-risk group were indicative of better immunotherapy response (*P* < 0.05).

**Conclusions:**

The novel signature offers a promising way to evaluate the prognosis and treatment efficacy in BC patients, which may facilitate clinical practice and management.

## 1 Introduction

Breast cancer (BC) is the most frequent malignancy in women, and its incidence rate increases by 0.5% annually (1, 2). In 2020, BC overtook lung cancer as the leading cause of cancer-related morbidity worldwide. In addition, it ranks fifth among global cancer-related deaths and places a great burden on society (3). Clinically, BC can be divided into four primary subtypes (4). Based on subtype classification, BC has distinct therapeutic strategies, including surgical intervention, radiotherapy, chemotherapy, endocrine therapy, and targeted therapy (5–7). However, after conventional treatment, approximately 25% BC patients develop distant metastases (8). In recent years, tremendous advances have been achieved in systemic treatment, and the long-term survival probability of BC patients has clearly improved. Immunotherapy is an emerging field in the management of BC, and multiple research has revealed that the immune system plays an indispensable role in the occurrence and development of BC (9). Previous studies have demonstrated that checkpoint inhibitors targeting programmed cell death-1/programmed death ligand-1 (PD-1/PD-L1) can effectively improve clinical outcomes in patients with advanced BC (10–12). Additionally, novel therapeutic strategies, including targeting myeloid-derived suppressive cells and regulatory T cells (Tregs), have attracted the attention of researchers (13, 14). However, currently, approximately 80% of patients fail to respond to these treatments due to tumor heterogeneity. Therefore, it is significant to explore the molecular characteristics of BC and identify biomarkers that can precisely predict its response to immunotherapy.

Metabolic reprogramming is an important hallmark of cancer that modulates energy metabolism in the tumor microenvironment, thus leading to the uncontrolled growth of cancer cells (15, 16). Recent studies have revealed that metformin, a widely used first-line drug for type II diabetes, has good efficacy against a variety of malignancies, including breast cancer (17). Therefore, we speculated that regulation of metabolism could be a promising strategy for tumor treatment. Nicotinamide (NAM) is the active amide form of vitamin B3 (18). It can be supplemented externally or synthesized *in vivo*. NAM is easily absorbed by various cells and serves as a precursor for the coenzyme nicotinamide adenine dinucleotide (NAD+). As an important enzyme in biological redox reactions, NAD+ participates in cellular energy metabolism and signal transduction, including the tricarboxylic acid cycle, DNA damage repair, and epigenetic regulation (19, 20). Previous studies have demonstrated that NAM supplementation effectively delayed aging (21). Senescence and cancer are interconnected. The reduction in NAD+ during cell senescence leads to an increase in reactive oxygen species (ROS), thus promoting the accumulation of hypoxia-inducible factor-1α, resulting in metabolic reprogramming. Based on these findings, researchers have begun to explore the antitumor potential of NAM. Previous studies have observed that NAM enhanced tumor blood flow and ameliorated the tumor hypoxia microenvironment, thus improving sensitivity to radiotherapy (22). Phase II trials in head and neck cancer and advanced bladder carcinoma have demonstrated the efficacy and safety of NAM as a radiosensitizer for radiotherapy (23, 24). In chronic lymphocytic leukemia, NAM exerted anticancer effects by inducing apoptosis *via* activation of the p53/miR-34a/SIRT1 pathway (25). In triple-negative BC, NAM regulated lipid metabolism and strengthened ROS-induced apoptosis-related pathways, thereby inhibiting tumor proliferation and invasion (18). Nicotinamide phosphoribosyl transferase (NAMPT), a key enzyme in NAD+ salvage synthesis, catalyzes NAM to generate nicotinamide mononucleotide (NMN) and is the initiating factor in the immunosuppressive microenvironment (26–28). In some tumors, NAMPT was found to drive PD-L1 expression and regulate tumor immune escape in a CD8+ T cell-dependent manner (20, 29). Based on these findings, we recognized the significance and predictive potential of NAM metabolism in tumor prognosis and immunity.

Therefore, in this study, we developed a six-gene NMRS using RNA-seq and clinical data from the TCGA database. Subsequently, we evaluated the predictive ability of the model for survival outcomes, immunotherapy response, and immune landscape. Moreover, we verified the expression of the gene signature at the transcriptional level through single-cell sequencing data (scRNA-seq).

## 2 Materials and methods

### 2.1 Data gathering

**Figure 1:**
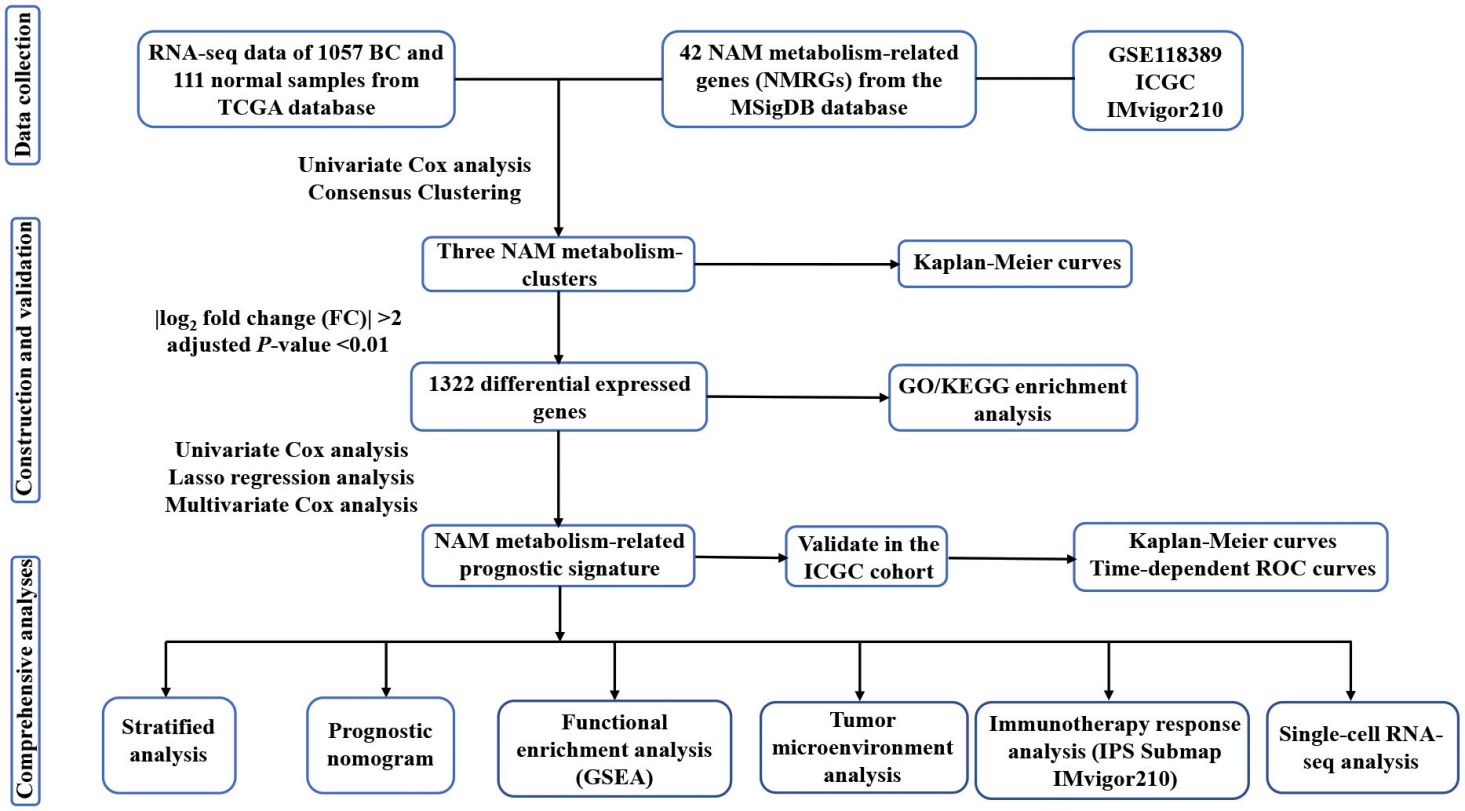
The flow chart of the study.

We collected RNA-seq data of 1057 BC samples and 111 adjacent normal samples from TCGA database (https://portal.gdc.cancer.gov/). TPM-formatted files for gene expression were acquired. The term TPM, which referred to transcripts per kilobase of exon model per million reads, indicated that it was successively normalized by the gene length and sequencing depth. Clinical data were obtained, including age, TNM stage, expression status of ER, PR, and HER-2, survival time and survival status. The prognostic prediction capacity of NMRS was verified using an external cohort made up of 98 BC samples that was retrieved from the ICGC database (https://dcc.icgc.org). Additionally, the IMvigor210 cohort, which included 298 advanced urothelial carcinomas with immunotherapy data, was obtained from the ‘IMvigor210CoreBiologies’ R package to predict therapeutic response (30). Moreover, scRNA-seq data (GSE118389) of 1534 BC cells were acquired from the GEO database (http://www.ncbi.nlm.nih.gov/geo) to validate the expression levels of the model genes in different cellular subtypes. The Molecular Signatures Database (MSigDB) was searched for two gene sets associated with NAM metabolism that were used in this investigation. The flow of the analyses is presented in **Figures 1**, **S1**.

### 2.2 Mutation landscape of NMRGs

Somatic mutation profiles were collected from TCGA database in the maf’ format. Using the ‘maftools’ R package, we plotted a waterfall diagram to visualize the mutation landscape of patients with BC. In addition, we obtained copy number variation (CNV) data from the UCSC Xena database (https://xena.ucsc.edu/) and analyzed the CNV frequency of the NMRGs.

### 2.3 Consensus clustering of the NMRGs

We utilized the ‘ConsensusClusterPlus’ R package to perform consensus clustering and identified distinct NAM metabolism-related clusters based on the expression level of the NMRGs (31). The cluster number (k) was set between 2 and 10, and the optimum k value was selected according to the cumulative distribution function. We evaluated the different clustering results and duplicated this procedure 1000 times to ensure stability and reliability. Furthermore, we used the ‘survminer’ R package to visualize the survival variations between different clusters.

### 2.4 Identification and enrichment analysis of the differentially expressed genes

Using the ‘limma’ package, differentially expressed genes (DEGs) were determined among different clusters. The screening criteria were |log_2_ fold change (FC)| more than 2 and an adjusted *P*-value of less than 0.01. Then, using the ‘clusterProfiler’ R package, Gene Ontology (GO) and Kyoto Encyclopedia of Genes and Genomes (KEGG) enrichment analyses for pathway and function annotations were conducted (32).

### 2.5 Development and validation of the NMRS

To derive the prognostic DEGs, a univariate Cox analysis was performed. After that, we ran a Lasso regression with a minimum penalty coefficient to avoid overfitting (33). We then conducted multivariate Cox analysis to identify the optimal independent predictive signature. The expression level and corresponding coefficient of each prognostic gene were used to calculate the risk score of BC patients: risk score = h_0_ (t)*exp[Σexpression*coefficient]. Based on the median cutoff value, we separated patients with BC into distinct risk groups for subsequent analyses. For the training, internal validation, entire TCGA and ICGC cohorts, survival curves were used to explore the ability of NMRS to differentiate prognosis between different risk groups. Similarly, the time-dependent receiver operating characteristic (ROC) curve was used to evaluate the reliability of the signature using the ‘timeROC’ package. Furthermore, we performed stratified analysis to assess the prognostic value of NMRS in distinct subgroups stratified by clinical characteristics.

### 2.6 Development and assessment of the NAM metabolism-related nomogram

We performed univariate and multivariate Cox regression of the 6-gene signature and clinical indicators to determine independent prognostic factors. Based on the independent predictors, we developed a nomogram to quantify the 3-, 5-, and 10-year survival probabilities of patients with BC. The consistency and accuracy of the nomogram were assessed using calibration and time-dependent ROC curves. Additionally, by using decision curve analysis (DCA), we evaluated the net benefit of the comprehensive nomogram versus the model that only included clinical variables.

### 2.7 Functional enrichment analysis

With the use of the annotated gene set ‘c2.cp.kegg.v7.5.1. symbols.gmt’ from the MSigDB database, we conducted gene set enrichment analysis (GSEA) to identify the variations in corresponding pathways between different risk groups (*P* < 0.05, false discovery rate (FDR) < 0.25) (34).

### 2.8 Identification of the immune landscape and immunotherapy efficacy

The ESTIMATE algorithm was used to evaluate the tumor purity, immune score, stromal score and ESTIMATE score (35). According to the ‘CIBERSORT’ algorithm, LM22 gene signature matrix was employed to measure the relative proportion of 22 immunocytes per sample under 1000 permutations (36–38). The relative infiltration of 28 immunocyte subpopulations in BC TME were quantified by single-sample gene set enrichment (ssGSEA) (39). The expression levels of a few checkpoint genes, the prevalence of tumor mutations, the score of the cancer-immunity cycle (CIC) and immunotherapy-related signals were then compared in different risk groups. Immunophenoscore (IPS) refers to the four major gene categories that determine immunogenicity, and is obtained by unbiased analysis using machine learning (40). Existing studies have confirmed that the IPS can be used as a predictive tool for the clinical outcomes of immunotherapy (41, 42). In this study, using IPS data from The Cancer Immunome Atlas (TCIA) (https://tcia.at/home), we evaluated the potential immunotherapy response between the high- and low-risk group. Additionally, the Submap algorithm (http://cloud.genepattern.org/gp) was applied to predict how the risk groups would react to anti-PD-1 and anti-Cytotoxic T-Lymphocyte Associated Antigen 4 (CTLA-4) therapy (43). To further evaluate the predictability of the NMRS for responsiveness to anti-PD-1 therapy, we downloaded the corresponding immunotherapy data and clinical information from the ‘IMvigor210CoreBiologies’ package for analysis.

### 2.9 Comparisons of drug sensitivity

Half-maximal inhibitory concentration (IC50) values for the most commonly used chemotherapeutic medicines were calculated using the ‘pRRophetic’ R package. The drug sensitivity of one risk group was compared to the other, and any statistically significant differences were tested using the Wilcox test (44, 45).

### 2.10 Validation of the 6-gene signature using scRNA-seq analysis

To further discriminate the cellular subtypes and illustrate the distribution of the model genes in various subtypes, we acquired an scRNA-seq cohort (GSE118389) from the GEO database for analysis. The ‘Seurat’ R package was used to convert the matrix into Seurat object and carry out strict quality control (46). The data were normalized and principal component analysis (PCA) was performed for genes with large coefficients of variation. Then, using the ‘SingleR’ package, t-distributed statistical neighbor embedding (tSNE) and subtype annotations were carried out to determine the model gene expression levels and internal relationships (47).

### 2.11 Statistical analyses

All statistical calculations and graphs were completed using R software 4.2.1. With the use of Kaplan-Meier curves and log-rank test, survival disparities between distinct risk groups were examined. In addition, we utilized the Wilcox test to evaluate the divergency between two sets of data. As a statistical threshold, a two-sided *P*-value <0.05 was used.

## 3 Results

### 3.1 Genomic and transcriptomic landscape of NMRGs

**Figure 2:**
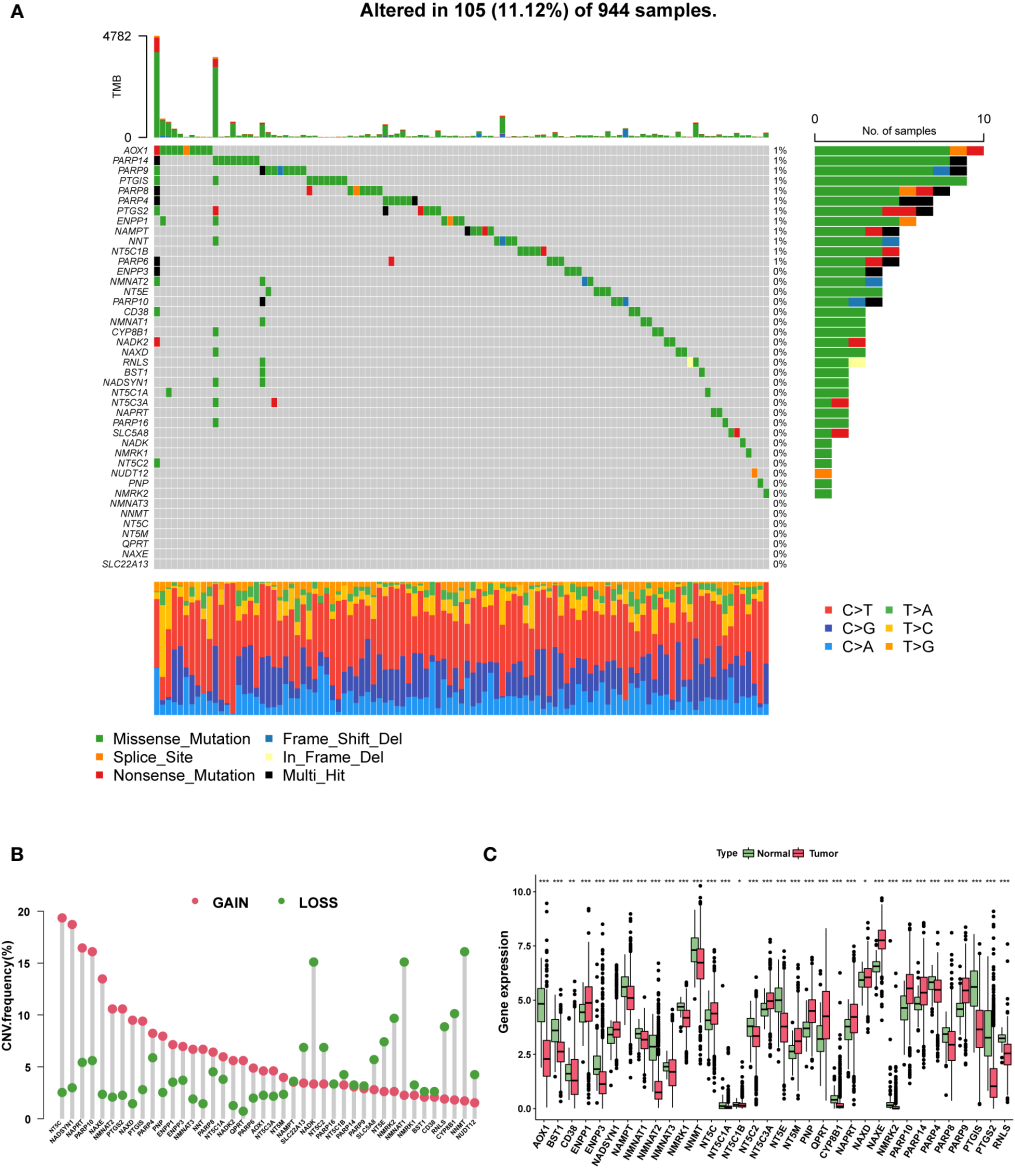
The genomic and transcriptomic landscape of NMRGs. **(A)** The mutation landscape of NMRGs in the TCGA database. **(B)** The CNV frequency of NMRGs. **(C)** The differential expression of NMRGs in BC between tumor and adjacent normal tissues (* p<0.05, ** p<0.01, *** p<0.001).

Based on the two NAM metabolism-related gene sets, we identified 42 NMRG (**Table S1**). In the genetic variation analysis, 105 (11.12%) of the 944 samples showed mutations, of which missense mutations were the most common (**Figure 2A**). The top three mutant NMRGs were Homo sapiens aldehyde oxidase 1 (AOX1), poly (ADPribose) polymerase family, member 14 (PARP14) and Homo sapiens poly (ADP-ribose) polymerase family, member 9 (PARP9). In addition, we analyzed the frequency of CNV in the NMRGs and observed that both amplification and loss of the copy number were frequent (**Figure 2B**). The differential analysis results demonstrated that, compared with the adjacent normal samples from TCGA database, there were 13 upregulated and 21 downregulated genes (*P* < 0.05) (**Figure 2C** and **Table S2**).

### 3.2 Consensus clustering analysis based on NMRGs

**Figure 3:**
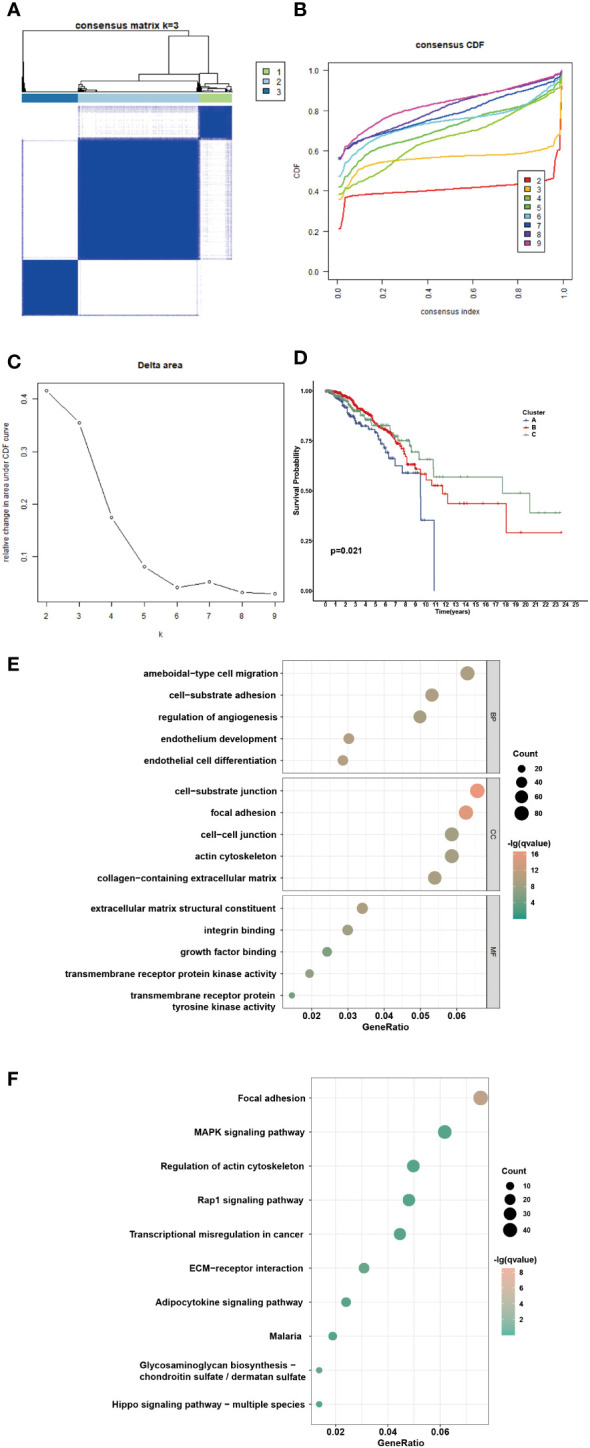
Identification of potential NAM metabolism-related clusters in BC patients. **(A)** The consensus clustering analysis of NMRGs in TCGA-BRCA cohort (k=3). **(B)** Consensus CDF. **(C)** Delta area. **(D)** The OS Kaplan-Meier curve of different clusters. **(E-F)** GO and KEGG enrichment analysis of differential expressed genes.

Consensus clustering was performed to identify the NAM metabolism-related clusters based on the expression levels of NMRGs. According to the cumulative distribution function, k = 3 exhibited excellent clustering resilience, with relatively strong intra-cluster correlation and low inter-cluster correlation (**Figures 3A–C**). As a result, patients were separated into three clusters: 168 patients placed in cluster A, 575 in cluster B, and 275 in cluster C. We observed that patients in cluster C had prolonged overall survival (**Figure 3D**, *P* = 0.021). Considering the significant differences in transcriptome levels and survival outcomes among the distinct clusters, we speculated about the presence of DEGs. According to the screening standard, we collected 1322 DEGs and then ran functional enrichment analysis on the DEGs. GO analysis enriched DEGs from three categories: biological processes (BPs), cellular components (CCs), and molecular functions (MFs). BPs were mainly enriched in ameboidal-type cell migration, cell-substrate adhesion, and regulation of angiogenesis. CCs were mainly enriched in cell-substrate junctions, focal adhesions, and cell-cell junctions. Additionally, the significantly enriched MFs were extracellular matrix structural constituents, integrin binding, and growth factor binding (**Figure 3E**). Results of the KEGG enrichment analysis showed that the DEGs were predominantly enriched in focal adhesion, the MAPK signaling pathway, and regulation of the actin cytoskeleton (**Figure 3F**). These findings suggested that the DEGs were involved in signaling pathway modulation and tumor growth.

### 3.3 Development and validation of the NAM metabolism-related prognostic signature

**Figure 4:**
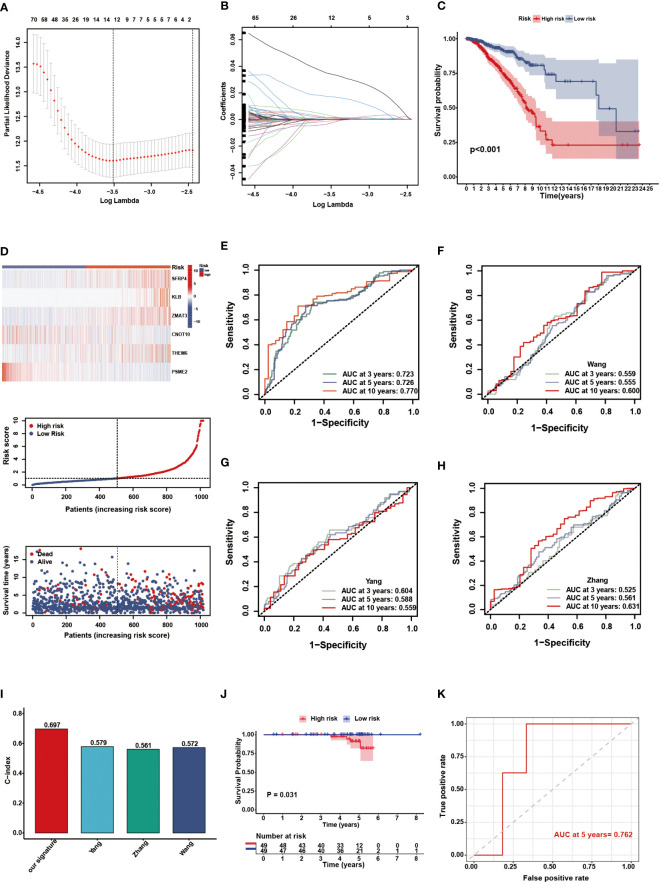
Construction and validation of the NMRS. **(A)** Cross validation method to select optimal genes. **(B)** The Lasso coefficient profiles. **(C)** The OS KM curves between high- and low-risk groups in the TCGA-BRCA cohort. **(D)** The model genes, risk score and clinical outcomes in the two risk groups. **(E)** The time-dependent ROC curves of the NMRS. **(F-H)** The time-dependent ROC curves of Wang’s, Yang’s and Zhang’s gene signature. **(I)** C-idex of our signature, Wang’s, Yang’s and Zhang’s signature. **(J)** The OS KM curves between high- and low-risk groups in the ICGC cohort. **(K)** The time-dependent ROC curves of the NMRS in the ICGC cohort.

Through univariate Cox analysis, we identified genes significantly associated with survival in patients with BC. Lasso regression demonstrated that the cross-validation effect was best when λ = -3.5, and relevant genes were included in the multivariate Cox analysis (**Figures 4A, B**). Finally, a NAM metabolism-related 6-gene signature was created to predict prognosis. The following equation was developed to calculate the risk scores based on gene expression levels and the regression coefficient: risk score = h_0_ exp [(0.002 × SFRP4) + (0.021 × KLB) + (0.051 × ZMAT3) – (0.022 × CNOT10) + (0.011 × C8orf55) – (0.008 × PSME2)]. Patients in the TCGA-BRCA cohort had their individual risk scores determined, and based on the median value, they were assigned to different risk groups. The survival curve showed that the low-risk group had a favorable OS compared with the patients in the high-risk group (**Figure 4C**, *P* < 0.001). The distribution of risk score, clinical outcomes, and model gene expression patterns between the two risk groups was displayed in **Figure 4D**. Among TCGA cohort, the AUC values for the predicted survival rates at 3-, 5-, and 10-year were 0.723, 0.726, and 0.770, respectively, demonstrating the robust prognostic power of the signature (**Figure 4E**). Compared with some existing BC biomarkers, our gene signature had better predictive performance with higher AUC and c-index values (**Figures 4F–I**) (48–50). Using a BC cohort from the ICGC database, we performed external validation to further confirm the predictive capacity of the NMRS. Based on the risk score calculation formula, 98 patients in the ICGC cohort were divided into high- (n = 49) and low-risk (n = 49) groups according to the median value. The KM survival curve showed that the survival of the high-risk group was significantly poorer than that of the low-risk group (**Figure 4J**, *P* = 0.031). Moreover, the AUC value of the 5-year OS in the external testing cohort was 0.762 (**Figure 4K**).

### 3.4 Stratified analysis and establishment of a NAM metabolism-based nomogram

**Figure 5:**
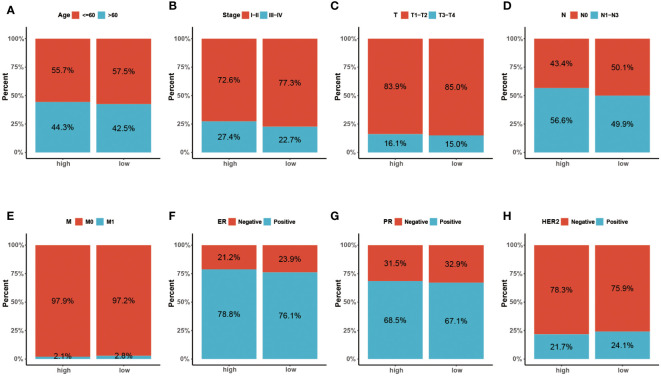
**(A-H)** The distribution characteristics of different clinicopathological factors in the two risk groups (Age, Stage, T, N, M, ER, PR and HER-2, respectively).

**Figure 6:**
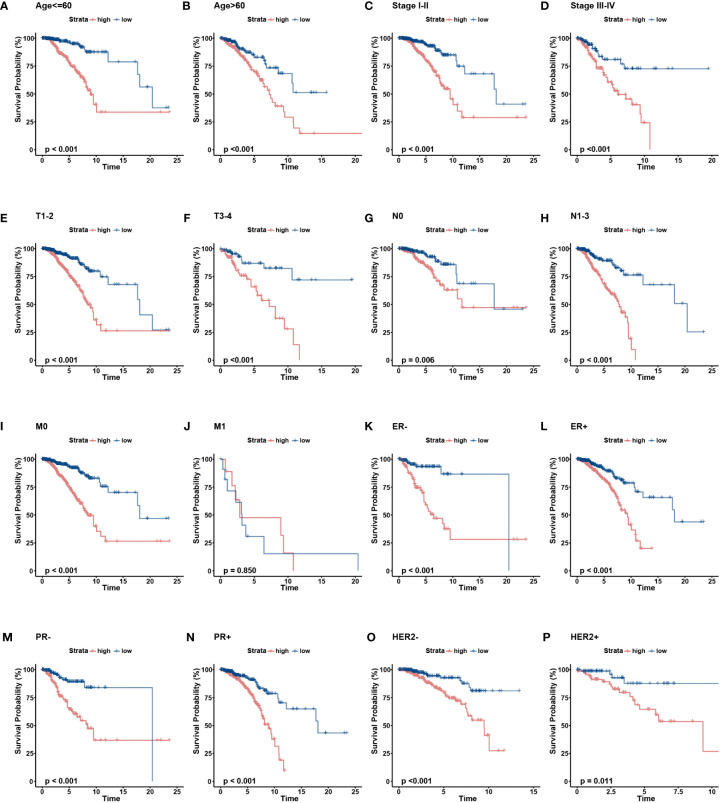
The OS KM curves of the two risk groups stratified by the clinicalpathological factors. **(A-B)** Age, **(C-D)** Stage, **(E-F)** AJCC T stage, **(G. H)**, AJCC N stage, **(I, J)** AJCC M stage, **(K, L)** ER status, **(M, N)** PR status, **(O, P)** HER-2 status.

**Figure 7:**
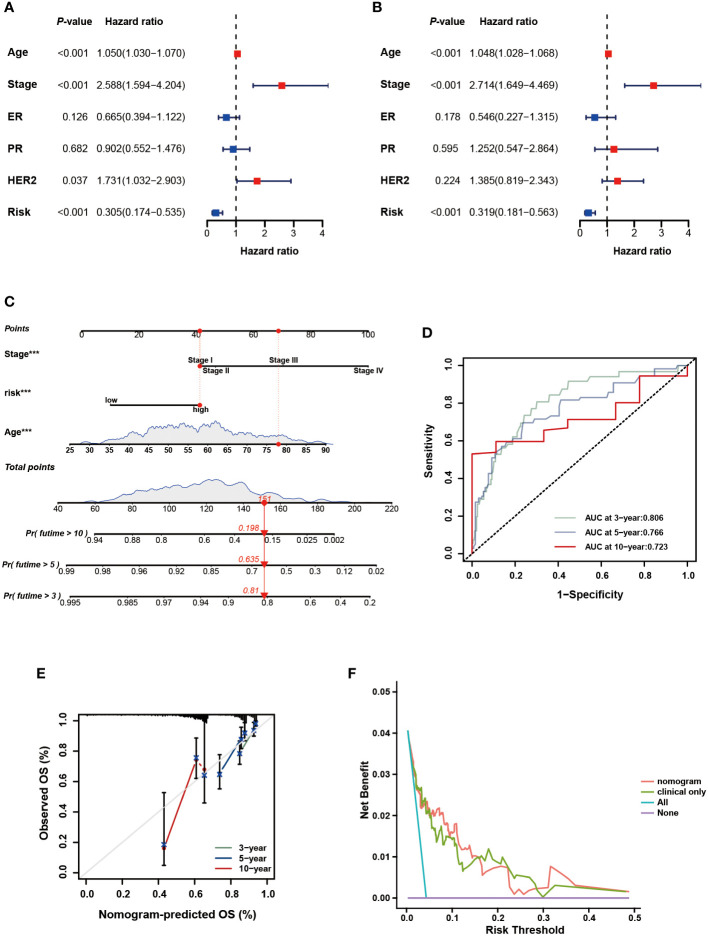
Development and assessment of the nomogram. **(A)** Univariate regression. **(B)** Multivariate regression of the clinicopathological indicators and gene signature. **(C)** A comprehensive nomogram for predicting BC patients’ survival probability. **(D)** The time-dependent ROC curves of the nomogram. **(E)** Calibration curves of the nomogram at 3-, 5-, and 10-year intervals. **(F)** DCA curves of the clinicopathological indicators and this nomogram.

To further verify the prognostic value of the signature in subgroups with distinct clinical features, we conducted a stratified analysis. Based on age, pathological stage, TNM stage, and ER, PR, and HER-2 status, we divided patients into different subgroups and performed survival analysis. **Figures 5A–H** showed the distribution characteristics of the different risk groups in each subgroup. The results showed that, consistent with the observation in the entire cohort, the low-risk group had better clinical outcomes in all subgroups except in the M1 stage (**Figures 6A–P**). Additionally, significant clinicopathological indicators and the gene signature were subjected to the univariate and multivariate Cox analyses. In the univariate regression analysis, we observed that age (HR: 1.050, 95% confidence interval (CI):1.030– 1.070, *P* < 0.001), stage (2.588, 1.594–4.204, *P* < 0.001), HER-2 status (1.731, 1.032–2.903, 0.037), and risk score (0.305, 0.174–0.535, *P* < 0.001) were significantly related to BC prognosis (**Figure 7A**). After adjusting for potential bias using multivariate regression analysis, age (1.048, 1.028–1.068, *P* < 0.001), stage (2.714, 1.649–4.469, *P* < 0.001), and risk score (0.319, 0.181–0.563, *P* < 0.001) were found to be independent predictors (**Figure 7B**). Based on the independent prognostic factors, a comprehensive nomogram was developed to make quantitative predictions of the 3-, 5-, and 10-year OS probabilities in patients with BC (**Figure 7C**). The AUC values were 0.806, 0.766, and 0.723 at 3, 5, and 10-years, respectively, indicating that the nomogram could provide accurate predictions (**Figure 7D**). The calibration curves demonstrated a high consistency between the predicted and actual OS (**Figure 7E**). The clinical applicability of the nomogram was examined using the DCA curve (51). Compared to the model with clinical characteristics only, we found that this comprehensive nomogram could generate more net benefits, which might contribute to better clinical management (**Figure 7F**).

### 3.5 Identification of the immune landscape

**Figure 8:**
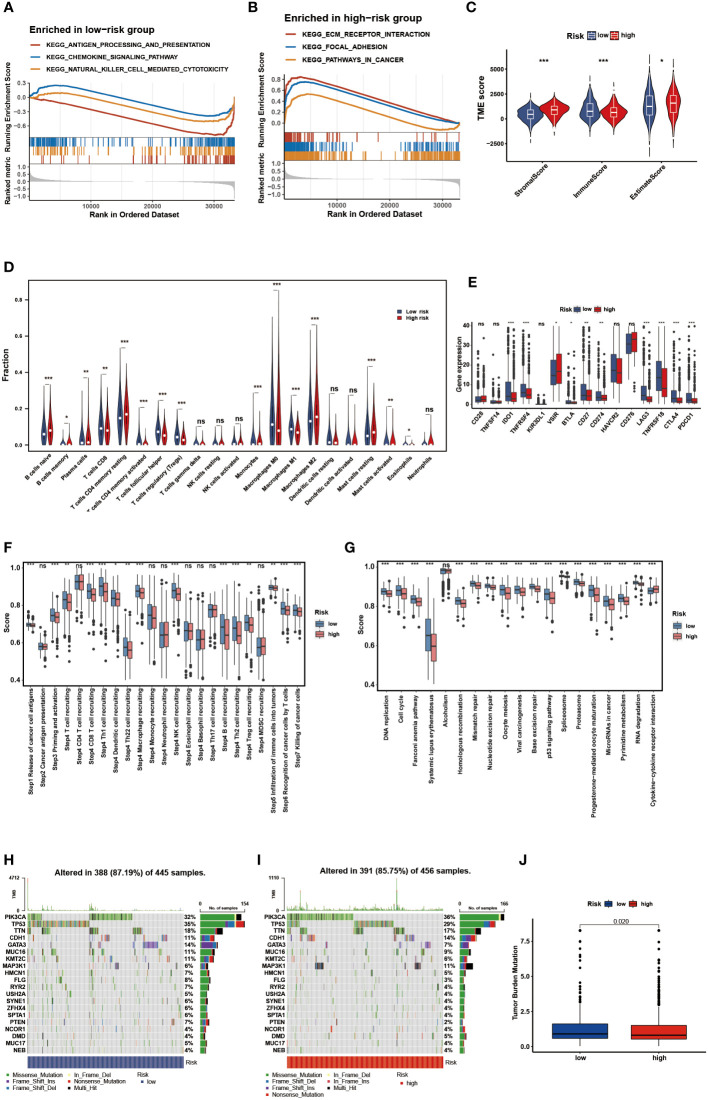
Identification of the immune landscape between the two risk groups. The GSEA of the DEGs. **(A)** in the low-risk group. **(B)** in the high-risk group. **(C)** Comparisons of the stromal, immune and ESTIMATE score in the two risk groups. **(D)** Comparisons of immunocyte’s infiltration fractions in the two risk groups. **(E)** The differential expression of checkpoint genes in the two risk groups. **(F)** Differences of cancer-immunity cycle scores in the two risk groups. **(G)** Differences of immune-associated positive signals in the two risk groups. The mutation landscape of the top 20 genes. **(H)** in the low-risk group. **(I)** in the high-risk group. **(J)** Comparisons of TMB in the two risk groups. * p<0.05, ** p<0.01, *** p<0.001, ns p>0.05.

GSEA of DEGs was performed to determine the biological processes of the two risk groups. Notably, the low-risk group was predominantly enriched in immune-associated processes, such as antigen processing and presentation, chemokine signaling pathways, and natural killer cell-mediated cytotoxicity, whereas the high-risk group was enriched in cancer-related processes, such as focal adhesion and ECM receptor interaction (**Figures 8A, B**). Then, the ESTIMATE and CIBERSORT algorithms were employed to explore the tumor microenvironment. ESTIMATE analysis showed that the low-risk group had lower stromal scores, estimated scores, and higher immune scores (*P* < 0.05) (**Figure 8C**). According to the results of CIBERSORT, the low-risk group had a significantly higher proportion of CD8 T cells, activated memory CD4 T cells, follicular helper T cells, regulatory T cells, M0 macrophages, M1 macrophages, activated mast cells, and eosinophils (*P* < 0.05) (**Figure 8D**). The ssGSEA results were demonstrated in a heatmap to visualize the relative abundance of 28 immunocyte subpopulations (**Figure S2**). We found that the immune infiltrating cell subpopulations with anti-tumor effects were mainly enriched in the low-risk group, such as the activated dendritic cell and activated CD4/CD8 T cell, while the immunocyte subpopulations with pro-tumor effects were mainly enriched in the high-risk group, such as the myeloid-derived suppressor cells (MDSC) and immature dendric cell. Additionally, nine frequent checkpoint genes, including the well-known BTLA, CTLA-4, and PDCD1, were strongly elevated in the low-risk group (*P* < 0.05) (**Figure 8E**). Furthermore, higher scores were observed in the committed steps of the cancer-immunity cycle and immune-associated positive signals in the low-risk group (*P* < 0.05) (**Figures 8F, G**). Tumor cells evade immunosurveillance by enhancing TMB, whereas TMB conversely serves as a predictor of immunological response. Thus, we further visualized the mutation landscape in distinct risk groups and explored the correlation between risk score and TMB. **Figures 8H, I** demonstrate the top 20 genes with high mutation frequency in the risk groups, of which PIK3CA and TP53 have been shown to be of great significance (52–55). 391 (85.75%) of the 456 samples had mutations in the high-risk group, whereas in the low-risk group, 388 (87.19%) of the 445 samples did so. Furthermore, significantly higher TMB was observed in the low-risk group than in the high-risk group (**Figure 8J**). These findings suggest that the immune landscape between the two risk groups differ considerably.

### 3.6 Prediction of immunotherapy efficacy

**Figure 9:**
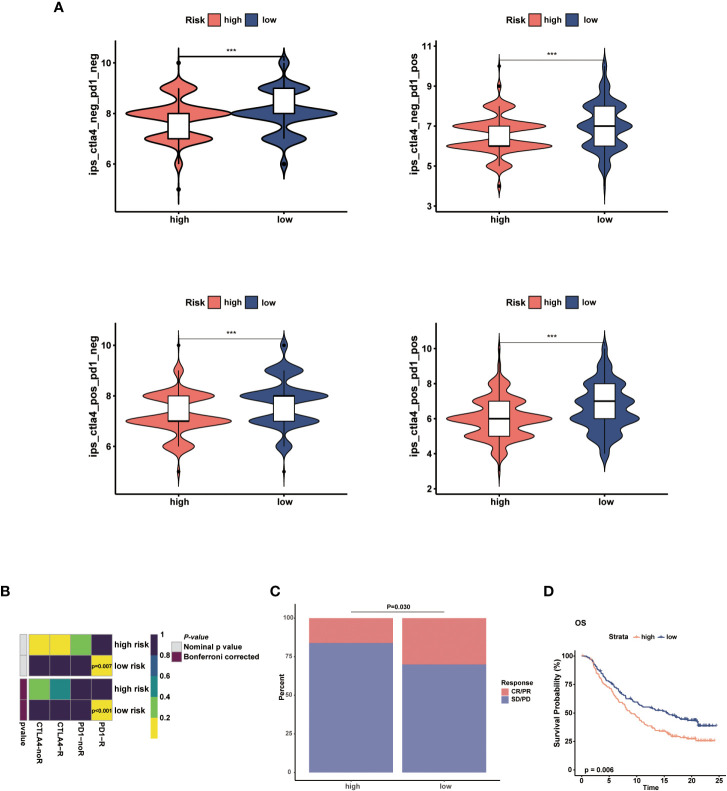
Prediction of the immunotherapy response in the two risk groups. **(A)** Comparisons of the IPS in the two risk groups. **(B)** Submap analysis between the two risk groups. **(C)** The proportion of clinical response to anti-PD-L1 immunotherapy in different risk groups in iMvigor210 cohort. **(D)** KM curves for the low-risk and high-risk groups in the iMvigor210 cohort. *** p<0.001.

The immunotherapy response in the two risk groups was evaluated using the IPS, Submap algorithms, and an external immunotherapy cohort. The IPS showed marked therapeutic benefits from checkpoint inhibitor treatment in the low-risk group (**Figure 9A**). Results from Submap revealed the therapeutic response to anti-CTLA4 and anti-PD-1 immunotherapy in BC patients (**Figure 9B**). The probability that the low-risk would react to anti-PD-1 immunotherapy was higher, as shown by the nominal *P*-value (*P* = 0.007) and Bonferroni corrected *P*-value (*P* < 0.001). Moreover, in the iMvigor210 cohort, we observed that the objective response rate (CR/PR) and survival probability in the low-risk group were higher than that in the high-risk group (**Figures 9C, D**). The outcomes in the external cohort verified that the NMRS could identify individuals that were immunotherapy-sensitive.

### 3.7 Comparisons of drug sensitivity

**Figure 10:**
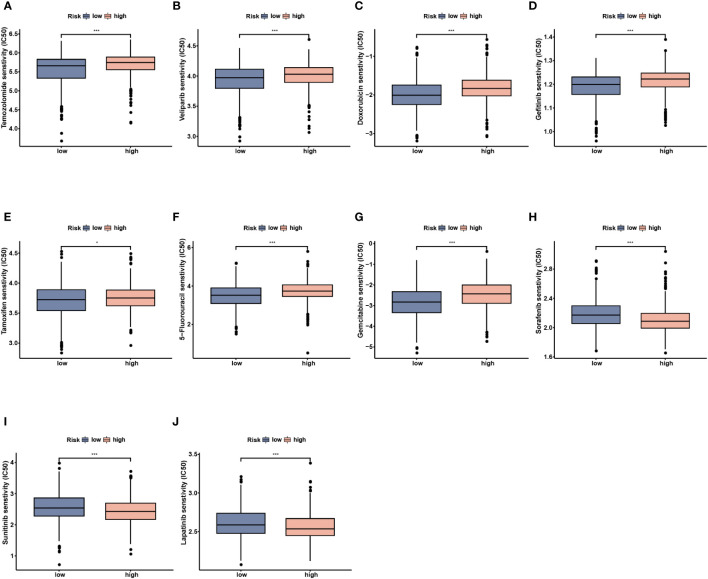
Drug sensitivity analysis between the low- and high-risk groups. **(A)** Temozolomide. **(B)** Veliparib. **(C)** Doxorubicin. **(D)** Gefitinib. **(E)** Tamoxifen. **(F)** 5-Fluorouracil. **(G)** Gemcitabine. **(H)** Sorafenib. **(I)** Sunitinib. **(J)** Lapatinib. * p<0.05, *** p<0.001.

To further investigate the clinical utility of NMRS in precise BC treatment, we assessed the therapeutic efficacy of frequently prescribed chemotherapeutic medications in different risk groups. According to the findings, low-risk individuals were more responsive to temzolomide, celiparib, doxorubicin, gefitinib, tamoxifen, 5-Flurouracil and gemcitabine, while less sensitive to sorafenib, sunitinib, and lapatinib (**Figures 10A–J**, *P* < 0.05).

### 3.8 Validation of the gene signature using scRNA-seq analysis

**Figure 11:**
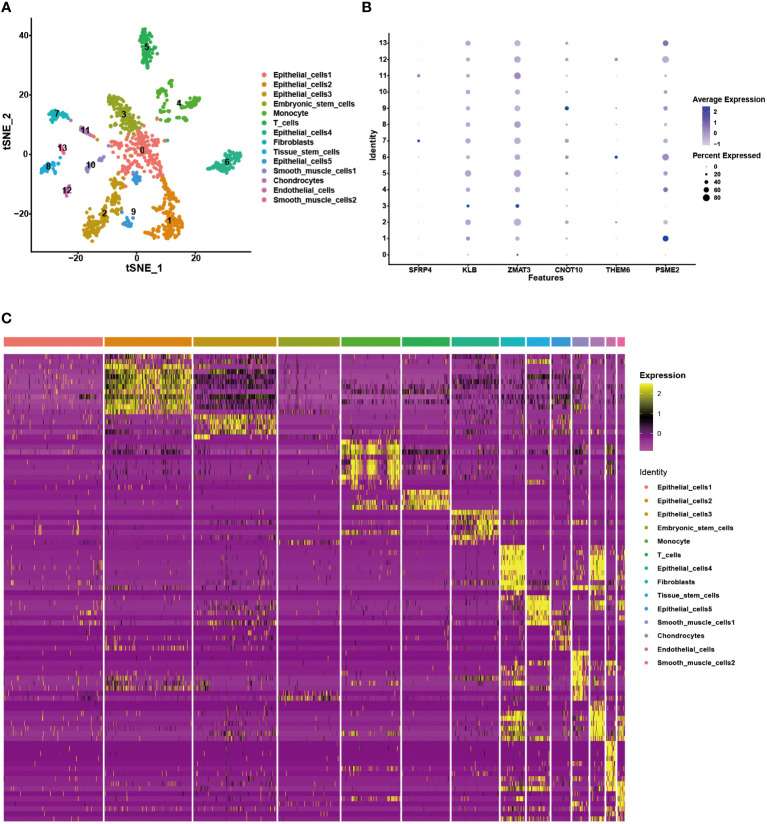
Validation of the gene signature in the scRNA-seq data. **(A)** The tSNE analysis showing 14 cellular subtypes in the scRNA-seq data. **(B)** The heatmap demonstrating the corresponding genes in each cellular subtype. **(C)** The expression level of the model genes in each cellular subtype.

We analyzed a scRNA-seq cohort to see whether the gene signature could be utilized to discriminate between different cellular subtypes. Using the tSNE analysis, fourteen clusters were identified (**Figure 11A**). These clusters could be divided into nine different cell subtypes, including epithelial cells, embryonic stem cells, monocyte, T cells, fibroblasts, tissue stem cells, smooth muscle cells, chondrocytes and endothelial cells, according to the results of the cellular subtype annotation (**Figure 11B**). The expression level of the gene signature in each subtype was displayed in a bubble plot, and the cellular subtypes could be distinguished (**Figure 11C**). KLB, ZMAT3, CNOT10, and PSME2 were highly expressed in most cell subtypes, whereas the other model genes had relatively specific expression patterns. SFRP4, and THEM6 were highly expressed in fibroblasts and epithelial cells, respectively. These findings demonstrated the stability of this gene signature for cellular subtype discrimination.

## 4 Discussion

Nicotinamide (NAM) is a water-soluble amide form of vitamin B3 and precursor of NAD+ (18). Components of NAM-related metabolism, such as NAD+, NMN, and the core enzyme NAMPT, play an important role in maintaining DNA repair and gene stability, and regulating the immune microenvironment (19, 20, 25–28). Recent studies have shown that NAM supplementation effectively inhibited the development of various malignancies such as breast cancer, chronic lymphocytic leukemia, and hepatocellular carcinoma (18, 25, 56). In recent years, the morbidity of BC has steadily increased, making it the most frequent malignancy in women. Clinicopathological characteristics (such as age, stage, histological grade, tumor size, and lymph node status) remain the primary indicators to predict prognosis and guide treatment in clinical practice. However, owing to the high heterogeneity of BC, traditional clinicopathological factors are insufficient to predict prognosis precisely. With the development of sequencing technology, an increasing number of researchers have begun to attach importance to the prognostic value of tumor molecular mechanisms, and corresponding biomarkers have been developed (57, 58). Several biomarkers have been applied in clinical molecular diagnosis and formulation of individualized treatment schemes. For example, the breast cancer 21-gene assay (Oncotype DX) is used to predict the recurrence risk and chemotherapy benefits in patients with hormone receptor-positive, HER2 receptor negative and lymph node-negative subtypes (59). Additionally, although some molecular risk models have not been used in clinical management, they show great potential for the precise prediction of BC, such as pyroptosis-associated and TP53 mutation-related models (60, 61). However, the significance of the NAM metabolism in BC remains unclear. In this study, we constructed a NAM metabolism-related signature to conduct risk stratification, predict prognosis, and provide immunotherapy guidance at the transcriptional level for clinicians treating patients with BC.

Due to the unrobustness of models based on individual genes, we identified a NAM metabolism-related 6-gene signature using machine learning methods. All of the six genes have significant roles in tumorigenesis and progression, but they are not correlated with each other. By combining the signature with two clinical indicators (age and pathological stage), a comprehensive nomogram was developed for accurate predictions. We observed that the risk score accounted for a considerable proportion of the total score in the model, verifying its significance.

Tumor microenvironment (TME) is a complex and dynamic ecosystem that mainly includes tumor cells, immune cells, and Sertoli cells. It plays a crucial role in the occurrence, development, and metastasis of tumors (62). Compared to the high-risk group, the low-risk group had a higher immune score, which was consistent with previous studies showing that high immune infiltration indicated a good clinical outcome (63–65). In addition, the low-risk group showed abundant infiltration of CD8 + T cells and M1 macrophages. Previous studies have reported that these immunocytes have robust anticancer and immunity-enhancing ability (66, 67). However, M2 macrophages and MDSCs were the primary component of the high-risk group immune cells. They can inhibit the immune response and promote tumor angiogenesis and lymphangiogenesis, thus leading to tumor growth and metastasis (68–70).

Tumor cells escape immune surveillance through multiple mechanisms, including activation of the immune checkpoint pathway. ICIs reverse immunological tolerance by overcoming tumor cell-mediated immune incapacity, restoring anticancer immunity, and clearing tumor cells (71). Previously, BC was not considered a highly immunogenic tumor owing to its low mutation burden and limited ability to form neoantigens (72). However, an increasing number of studies have reported a close association between BC and the immune system. Turajlic et al. found that, compared with other subtypes, TNBC has a relatively high TMB that can lead to an increase in tumor-related antigens, making it possible for the immune system to recognize and fight against tumor cells (73). Su et al. observed that trastuzumab treatment in HER2-positive patients can reshape the TME and enhance PD-L1 expression, providing a theoretical basis for the combination of immunotherapy and targeted therapy (74). Researchers have also found that the combination of endocrine therapy with ICIs may cause a decline in immunosuppressive cells in hormone receptor (+) patients (75). In summary, patients with all BC subtypes may benefit from immunotherapy. However, the efficacy of immunotherapy varies greatly among individuals, and only a portion of patients can benefit from it (76). Therefore, the development of predictive biomarkers for ICI treatment is particularly important for screening specific populations for individualized treatment. Currently, some biomarkers for predicting treatment response to ICIs have been identified, such as tumor mutation burden and CD8 infiltration (77, 78). In this study, based on the NAM metabolism-related signature, we used multiple algorithms and an independent cohort to explore the immunotherapy response between different risk groups. The results consistently showed patients in the low-risk group were more likely to benefit from immunotherapy, demonstrating the signature’s robust predictive power for immunotherapy response. In addition, a comprehensive consideration of this gene signature could effectively distinguish distinct BC cell subtypes, showing great application prospects.

However, our study had some limitations. First, our research was based on an existing public database and the findings require multicenter prospective trials for validation. Second, there may be some unknown interactions between genes and gene products in the signature, which has implications in physiology and pathology. Further exploration is required to characterize the mechanisms of the identified gene signature *in vitro* and *vivo* experiments.

## 5 Conclusion

In summary, we identified a novel NAM metabolism-related signature for the prognostic prediction in BC using bioinformatic analyses. Moreover, the gene signature had promising potential for predicting the immune microenvironment and immunotherapy response, which might facilitate clinical management.

## Data availability statement

Publicly available datasets were analyzed in this study. This data can be found here: https://portal.gdc.cancer.gov/, https://dcc.icgc.org, http://www.ncbi.nlm.nih.gov/geo, and https://xena.ucsc.edu/.

## Author contributions

HK and XM designed the study and supervised the completion, HC and XR contributed to data collection and analysis, HC, LD and LC wrote the manuscript, DL and ZZ reviewed the background and edited the manuscript. All the authors approved the final version of the manuscript.

## Supplementary material

Supplementary Figure 1 The workflow of this study.

Supplementary Figure 2 Single-sample gene set enrichment analysis identified the relative infiltration of 28 types of immune cell subpopulations in different risk groups.

Supplementary Table 1 A total of 42 NAM metabolism-related genes used in this study

Supplementary Table 2 The upregulated and downregulated NAM metabolism-related genes.
