# Supplementary figures and images for: Comprehensive analysis of nicotinamide metabolism-related signature for predicting prognosis and immunotherapy response in breast cancer
Source: Front Immunol. 2023 Mar 8;14:1145552. doi: 10.3389/fimmu.2023.1145552 (PMC10031006; doi:10.3389/fimmu.2023.1145552)

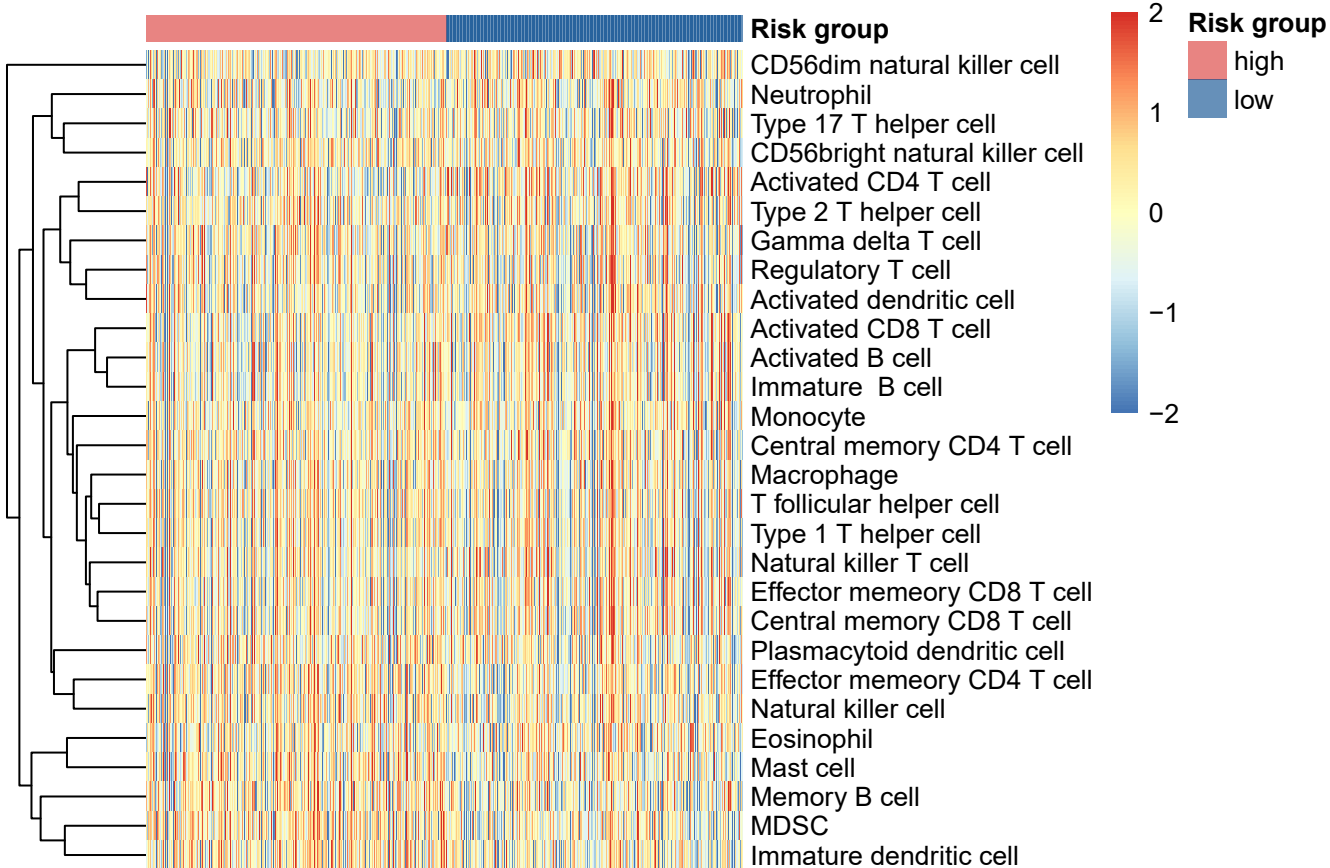

Supplement: Supplementary Figure 1 — The workflow of this study. [file DataSheet_1.pdf]
