# Supplementary material for: Comprehensive analysis of nicotinamide metabolism-related signature for predicting prognosis and immunotherapy response in breast cancer
Source: Front Immunol. 2023 Mar 8;14:1145552. doi: 10.3389/fimmu.2023.1145552 (PMC10031006; doi:10.3389/fimmu.2023.1145552)
Supplement: Supplementary Table 1 — A total of 42 NAM metabolism-related genes used in this study [file Table_1.docx]

| **NAM metabolism-related genes** | **NAM metabolism-related genes** |
| --- | --- |
| AOX1 | NUDT12 |
| BST1 | PNP |
| CD38 | QPRT |
| ENPP1 | CYP8B1 |
| ENPP3 | NADK2 |
| NADK | NAPRT |
| NADSYN1 | NAXD |
| NAMPT | NAXE |
| NMNAT1 | NMRK2 |
| NMNAT2 | PARP10 |
| NMNAT3 | PARP14 |
| NMRK1 | PARP16 |
| NNMT | PARP4 |
| NNT | PARP6 |
| NT5C | PARP8 |
| NT5C1A | PARP9 |
| NT5C1B | PTGIS |
| NT5C2 | PTGS2 |
| NT5C3A | RNLS |
| NT5E | SLC22A13 |
| NT5M | SLC5A8 |
