# Supplementary material for: Comprehensive analysis of nicotinamide metabolism-related signature for predicting prognosis and immunotherapy response in breast cancer
Source: Front Immunol. 2023 Mar 8;14:1145552. doi: 10.3389/fimmu.2023.1145552 (PMC10031006; doi:10.3389/fimmu.2023.1145552)
Supplement: Supplementary Table 2 — The upregulated and downregulated NAM metabolism-related genes. [file Table_2.docx]

| **Downregulated genes** | **Upregulated genes** |
| --- | --- |
| AOX1 | ENPP1 |
| BST1 | NADSYN1 |
| CD38 | NT5C |
| ENPP3 | NT5C3A |
| NAMPT | NT5M |
| NMNAT1 | PNP |
| NMNAT2 | QPRT |
| NMNAT3 | NAPRT |
| NMRK1 | NAXD |
| NNMT | NAXE |
| NT5C1A | PARP10 |
| NT5C1B | PARP14 |
| NT5C2 | PARP9 |
| NT5E |  |
| CYP8B1 |  |
| NMRK2 |  |
| PARP4 |  |
| PARP8 |  |
| PTGIS |  |
| PTGS2 |  |
| RNLS |  |
