# Supplementary material for: Comprehensive analysis of nicotinamide metabolism-related signature for predicting prognosis and immunotherapy response in breast cancer
Source: Front Immunol. 2023 Mar 8;14:1145552. doi: 10.3389/fimmu.2023.1145552 (PMC10031006; doi:10.3389/fimmu.2023.1145552)
Supplement: Supplementary Figure 2 — Single-sample gene set enrichment analysis identified the relative infiltration of 28 types of immune cell subpopulations in different risk groups. [file DataSheet_2.pdf]

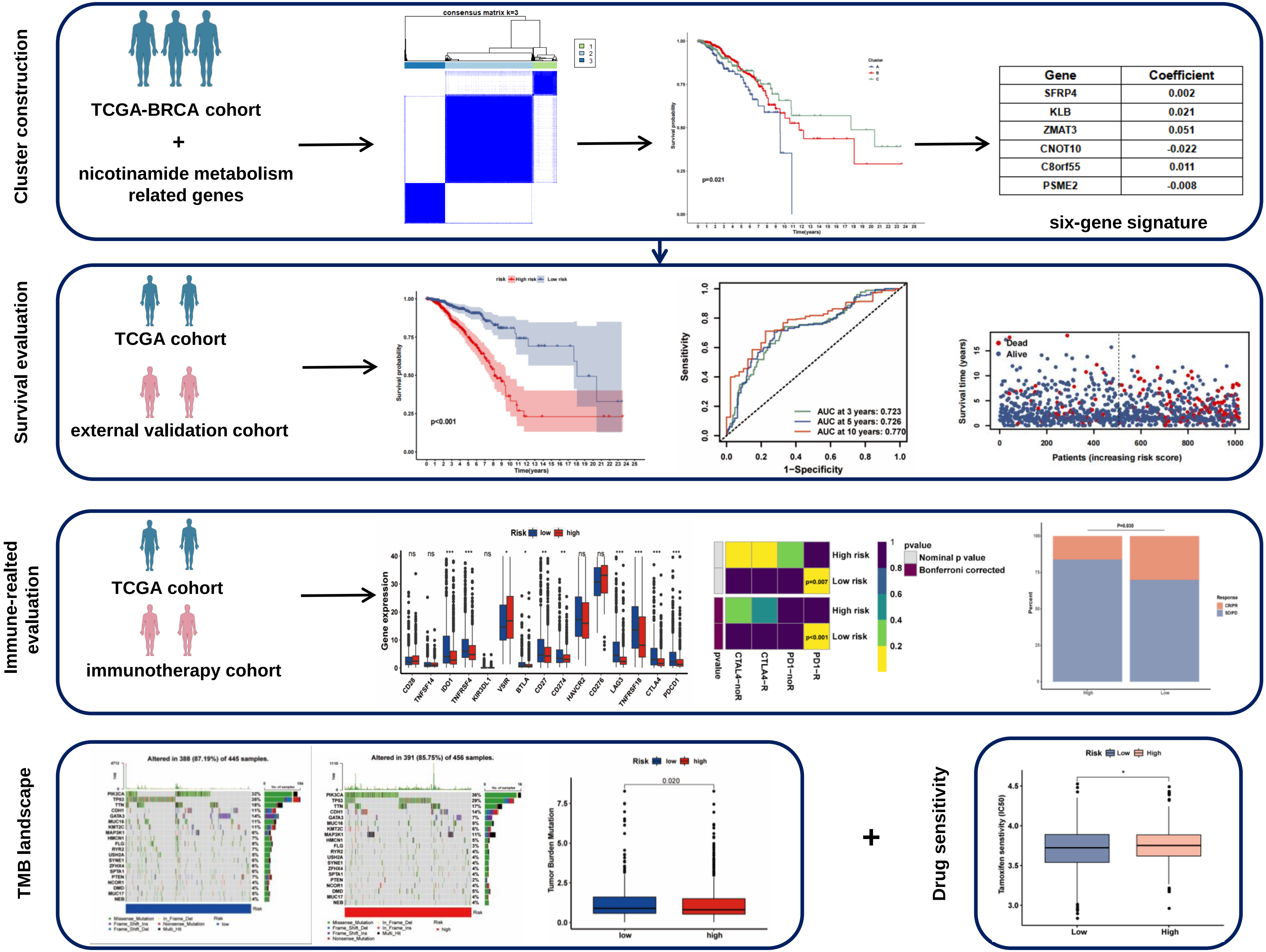
Cluster construction
TCGA-BRCA cohort
+
nicotinamide metabolism related genes
consensus matrix k=3
p=0.021
Time(years)
Survival probability
Cluster
Gene
Coefficient
SFRP4
0.002
KLB
0.021
ZMAT3
0.051
CNOT10
-0.022
C8orf55
0.011
PSME2
-0.008
six-gene signature
Survival evaluation
TCGA cohort
external validation cohort
risk
High risk
Low risk
p<0.001
Sensitivity
1−Specificity
AUC at 3 years: 0.723
AUC at 5 years: 0.726
AUC at 10 years: 0.770
Dead
Alive
Survival time (years)
Patients (increasing risk score)
Immune-realted evaluation
TCGA cohort
immunotherapy cohort
Risk
low
high
Gene expression
CD28
TNFSF14
IDO1
TNFRSF4
KIR3DL1
VSIR
BTLA
CD27
CD274
HAVCR2
CD276
LAG3
TNFRSF18
CTLA4
PDCD1
High risk
Low risk
p=0.007
p<0.001
pvalue
Nominal p value
Bonferroni corrected
CTLA4−noR
CTLA4−R
PD1−noR
PD1−R
P=0.030
Percent
Response
CR/PR
SD/PD
High
Low
TMB landscape
Altered in 388 (87.19%) of 445 samples.
Altered in 391 (85.75%) of 456 samples.
Risk
low
high
0.020
Tumor Burden Mutation
+
Drug sensitivity
Risk
Low
High
Tamoxifen senstivity (IC50)
